# Clinical Holistic Medicine: When Biomedicine is Inadequate

**DOI:** 10.1100/tsw.2004.32

**Published:** 2004-05-19

**Authors:** Søren Ventegodt, Mohammed Morad, Eytan Hyam, Joav Merrick

**Affiliations:** ^1^ The Quality of Life Research Center, Teglgårdstræde 4-8, DK-1452 Copenhagen K, Denmark; ^2^ Division of Community Health, Ben Gurion University, Beer-Sheva, Israel; ^3^ Soroka University Medical Center, Clalit Health Services, Faculty of Health Sciences, Ben Gurion University, Beer-Sheva, Israel; ^4^ National Institute of Child Health and Human Development, Office of the Medical Director, Division for Mental Retardation, Ministry of Social Affairs, Jerusalem and Zusman Child Development Center, Division of Pediatrics and Community Health, Ben Gurion University, Beer-Sheva, Israel

**Keywords:** quality of life, QOL, philosophy, human development, holistic medicine, public health, family medicine, Denmark, Israel

## Abstract

The modern physician is using pharmaceuticals as his prime tool. Unfortunately, this tool is much less efficient than you might expect from the biochemical theory. The belief in drugs as the solution to the health problems of mankind, overlooking important existing knowledge on quality of life, personal development, and holistic healing seems to be one good reason why approximately every second citizen of our modern society is chronically ill. The biomedical paradigm and the drugs are certainly useful, because in many situations we could not do without the drugs (like antibiotics), but curing infections or diseases in young age is not without consequences, as the way we perceive health and medicine is influenced by such experiences. When we get a more severe disease in midlife, we also believe drugs will make us healthy again. But at this age, the drugs do not work efficiently anymore, because we have turned older and lost much of the biological coherence that made us heal easily when we were younger. Now we need to assume responsibility, take learning, and improve our quality of life. We need a more holistic medicine that can help us back to life by allowing us to access our hidden resources. The modern physician cannot rely solely on drugs, but must also have holistic tools in his medical toolbox. This is the only way we can improve the general health of our populations. Whenever NNT (Number Needed to Treat) is 2 or higher, the likelihood of the drug to cure the patient is less than 50%, which is not satisfying to any physician. In this case, he must ethically try something more in order to cure his patients, which is the crossroads where both traditional manual medicine and the tools of a scientific holistic medicine are helpful.

## INTRODUCTION

About one in two persons have a chronic disease — unpleasant complaints such as arthritis, migraine, allergy, diabetes, or low back pain — despite numerous visits to their family physician or to specialists and generous use of advanced biomedicine[1]. About half the patients even seek alternative treatment[2], which is gaining increasing trust among the population, but often to no avail — the disease usually does not go away. In our modern society, partly due to longer life expectancy, illness has become something that people have to learn to live with. Often we see one disease followed by another and it is not uncommon for older people to have five or ten different ailments taking up to a dozen different kinds of medications. We also observe a steady decline in functional capacity of middle-aged or elderly people, who rely solely on biomedicine.

One of the most brutal tendencies of the biomedical paradigm[3] is that it often creates resignation and thus allows disease to control the individual. Symptoms can often be alleviated to some extent, but the causes of the disease are beyond the reach of acknowledgment due to the inherent philosophy of the biomedical paradigm: the overwhelming complexity of the biochemical description of man. Therefore, biomedicine is rarely able to help people get rid of the cause of their disease, which in our understanding often is to be found in the quality of the life the patients lead, and not in their genes or metabolism. The diseases plaguing our patients are often caused by lifestyle.

In our opinion, modern biochemistry generally is incapable of helping people to draw on their hidden resources. Thus, the human resources that should help us overcome the disease remain hidden. That is our harshest criticism of biomedicine as holistically oriented physicians. On the pretext of being able to help, it takes responsibility for and away from patients and thereby deprives them of the opportunity to wake up and help themselves — and patients let the physician do so. The consciousness-based medicine that we are striving to develop serves the opposite purpose, namely to help people help themselves. Many people have good experiences with biomedical treatment, since we all have had an infection or ailment at some time that was cured. With all its technological perfection and scientific character, biomedicine appears extremely convincing and makes us confident that it will provide a cure the next time something goes wrong. Unfortunately, the bitter truth is that once you get seriously ill, you usually remain ill for the rest of your life. Biomedicine cannot make you well again or cure the chronic illness.

Below, we provide some examples of quite common diseases and disorders for which biomedicine, in our experience as physicians, has proven inadequate. A colleague with more experience and greater insight in biomedicine might possibly be able to do a better job, so we will let readers draw their own conclusions on the numerous short and somewhat sad case histories. Before we present these case records, it is only fair to the reader to give a short introduction to conscious-based, holistic medicine.

## CLINICAL HOLISTIC MEDICINE

The life mission theory[4,5,6,7,8,9] states that everybody has a purpose of life, or a huge talent. Happiness comes from living this purpose and succeeding in expressing the core talent in your life. To do this, it is important to develop as a person into what is known as the natural condition, a condition where you know yourself and use all efforts to achieve what is most important for you. The holistic process theory of healing[10,11,12,13] and the related quality of life theories[14,15,16] state that the return to the natural state of being is possible, whenever the person gets the resources needed for existential healing. The resources needed are "holding" in the dimensions: awareness, respect, care, acknowledgment and acceptance with support and processing in the dimensions: feeling, understanding, and letting go of negative attitudes and beliefs. The preconditions for holistic healing to take place is the physicians' love of their patients, inviting the patients to trust, and when the patients trust their physicians, they can surrender and receive the holding that is a prerequisite for entering the special state of consciousness that we call "being in the holistic process of healing". Existential healing is not a local healing of any tissue, but a healing of the person as a whole, making him much more resourceful, loving, and knowledgeable of himself, his own needs and wishes. In letting go of negative attitudes and beliefs, the person returns to a more responsible existential position and an improved quality of life. The philosophical change of the person healing is often a change towards preferring difficult problems and challenges, instead of avoiding difficulties in life[17,18,19,20,21,22,23,24]. The person who becomes happier and more resourceful often also becomes more healthy, more talented, and more able to function[25,26,27].

## CHRONIC DISORDERS

The following case reports describe chronic disorders and complaints for which biomedicine has no effective remedy or cure, as is so often the case.

> **Female, aged 21 years, with chronic dizziness.** The patient suffers from chronic dizziness. Blood chemistry normal, except CRP (C-reactive protein), which is marginally outside the normal range. Against this background, we discuss whether the patient may have another illness that would explain the dizziness, e.g., a virus affecting the acoustic nerve. The patient is told to return if the symptoms do not go away by themselves.

Dizziness is more frequently encountered in older persons with increasing age. Most cases are benign and self-limited, but still a risk factor for falls in the older patient. Dizziness with no apparent organic cause is common and from a biomedical perspective very little can be done about it. “A virus affecting the acoustic nerve,” says the biomedical doctor, which is the explanation given to the patient to excuse why we cannot help, because there is not much we can do about a virus. Indeed, the dizziness will very often cease within 6 months.

However, this story might look different in a holistic perspective. Frequently, the dizziness either passes quickly or it continues for the rest of the patient’s life. In either case, it is unlikely to be caused by a virus. We believe that this kind of dizziness is generally caused by a loss of vital energy due to inner conflicts and lack of confrontation with both the internal and external reality. When the patient does not have enough energy to obtain an overview of life, this kind of dizziness occurs, like motion sickness. It simply becomes difficult to orientate oneself in the world. Once the problems tied down the vital energy, but when this conflict is solved, the dizziness will miraculously vanish.

> **Male, aged 25 years, with chronic sore throat.** No fever, sore throat for 4 weeks. Also pricking sensation in the tongue. Oral cavity: slightly red and swollen, no coating. Glands in the sternoclavicular region swollen bilaterally. Strep A: negative. To be reassessed in 2 weeks. Pricking sensation in the tongue possibly due to allergy. Prescribe Zyrtec [cetirzine] for the patient to try.

There is no effective treatment against a chronic burning and pricking sensation in the tongue and throat when there is no external cause. In our opinion, irritation of the tissue — a burning, hot and pricking sensation — is a clear sign of a blockage or hidden feelings. Symptoms can be alleviated by antihistamines and the effect may last for a few hours, but in the long term this is not a lasting solution. The patients suffer their entire lives. Sometimes they “grow” out of it, sometimes their illness takes on a more serious nature.

> **Female, aged 42 years, with tinnitus.** Noise in the ear, so that the patient finds it difficult to talk to other people. /Tinnitus/ Prescribe audiometry prior to assessment of the need for hearing aid with masking device. Note: Tinnitus may decrease if the patient’s possible depression improves, the picture of this suspected depression is unfortunately not so clear that is justifies a treatment. Hearing test shows nearly complete loss of hearing at high frequencies (over 2000 Hz). Referred to audiologist for hearing aid with masking device.

Tinnitus is a term to describe an internal noise perceived by the person. The cause can be otologic, metabolic, neurologic, pharmacologic, dental, or psychologic, but also due to vascular abnormalities, tympanic muscle disorders, or central nervous system anomalies. A complete otolaryngeal evaluation and audiometry should be performed in order to find the potential cause. Tinnitus is a very difficult problem to treat, with a prevalence of 14.2% in Gothenburg[28], thought to be representative for the Nordic countries.

## OBESITY

Childhood, adolescent, and adult obesity presents one of the most challenging and frustrating problems in medical practice. Obesity is of particular concern because of the health risks associated with it. These risks include hypertension, hyperlipidemia, hypertriglyceridemia, diabetes mellitus, coronary heart disease, pulmonary and renal problems, surgical risks, and degenerative joint disease. Obesity causes significant morbidity as well as a decreased life expectancy. Obesity is a major public health problem. Recent data from the Third National Health and Nutrition Examination Survey (NHANES) in the U.S. suggested that 22% of children and adolescents are overweight and 11% are obese. Among the general population, the survey revealed that between 1987 and 1993, overweight prevalence increased 3.3% for men and 3.6% for women. Total overweight prevalence among American males is now 33% and for females 36%[29].

**Female, aged 30 years, severely overweight.** The patient weighs 99 kg, BMI 44.5 = severe overweight (obese class III). Headache and tingling sensation in left hand. Also many episodes of reflux. Gastroscopy should be considered if the problem persists. Patient must return for weight-loss plan.

This patient needs a *life***.** Slight tingling sensation in her hand. Our guess is that she has more important issues to worry about. Why focus on the hand, when her entire body needs to be reviewed? And why look only at the body when her entire life has gone off track? This might seem a hard judgment, but in our opinion, a weight-loss plan or a reduction in calorie intake for that matter would be of little help to her, as she no doubt already has tried all dietary cures on the market to lose weight. To conquer her obesity, she has to address the fundamental problems, of which her obesity is a result.

The real weakness of biomedicine is that it does not support the patient in taking responsibility for his or her own life; this weakness is not only a fault of biomedicine as it is shared by the majority of alternative treatments. It is often the case that the person (physician or alternative healer) giving treatment does something with the patient on the basis of the *own* knowledge of the physician or healer. Physicians are often highly skilled and it is tempting to take control and know what is right. However, that is generally not the best possible help for the patient. The best result will not be achieved until the patient plays an active part to uncover his or her basic needs and finds out how to fulfill them.

## WHEN PAIN PERSISTS

Acute pain is a normal sensation triggered in the nervous system to alert you to possible injury and the need to take care of yourself, but chronic pain is different. Chronic pain persists. Pain signals keep firing in the nervous system for weeks, months, even years. There may have been an initial mishap — sprained back, serious infection — or there may be an on-going cause of pain — arthritis, cancer, ear infection — but some people suffer chronic pain in the absence of any past injury or evidence of body damage. Many chronic pain conditions affect older adults. Common chronic pain complaints include headache, low back pain, cancer pain, arthritis pain, neurogenic pain (pain resulting from damage to the peripheral nerves or to the central nervous system itself), or psychogenic pain (pain not due to past disease or injury or any visible sign of damage inside or outside the nervous system). One Dane in five lives a life of chronic pain in spite of the most effective biomedical treatment[3]. The pain persists despite analgesics, despite physiotherapy and massage, or despite antidepressants. The patient is in pain, regularly or more or less constantly, and life is not fun any more.

Pain is what causes almost 30% of the patients to see their family physician[30] and often the physician does not succeed in removing the pain from the everyday life of the patient. We do not want to hand out morphine to people who are not terminally ill, although that would be an effective pharmacological solution to both physical and existential pain. This could be one of the reasons why we have so many drug addicts or so many young girls in existential pain who become drug-addicted prostitutes. Let us make it clear that we do not share the restrictive attitude of our society to morphine. If it were up to us to decide, all adults would be allowed to buy it at the pharmacy. That would spare thousands of young people from humiliation, criminalization, marginalization, prostitution, or HIV and society would save enormous amounts of money. The way we see it, the drug policy of our society reflects old moral codes and notions, instead of being an expression of real insight into human suffering.

> **Male, aged 65 years, with chronic pain, reduced vitality, libido, and urge for isolation.** Patient has had failing health with many complaints of pain over the last 4 years. He has gone through several assessments with X-rays of knees and hips, with only minimum findings. In recent years, he has had decreased vitality and libido, now urge for isolation — mostly stays at home and indoors. Born in Asia. Has lived in Denmark for 30 years, but with a 4-year stay abroad in between. Denies any problems regarding language and culture. Examination: Knees almost normal findings, in particular no restricted movement, no looseness, no signs of arthritis, no patella effusion or other pathology. The patient reports slight tenderness on side of right knee. Hip also normal regarding movement. The patient complains of some pain in the extreme position [fully extended or flexed], but this is hardly relevant during normal use of the body. Below the note from the last radiological examination showing stable conditions in the patient's joints: X-ray of left hip joint compared with the right hip joint, shows, as previously, slight narrowing of the joint space on both sides, slightly more on the right than on the left. No deformation of femoral head/incipient osteoarthrosis of the hip bilaterally/.

This patient's situation is deteriorating. But what is actually the matter with him? The cultural problems appear to be insurmountable, but he denies them completely. This is a case of marginalization, perhaps even social exclusion. He is experiencing an existential desert, deep despair over no longer being useful. We are convinced that this man's situation in life could be rescued if he acknowledges, however painfully, the actual nature of his problems. But that is a barrier only he himself can cross. And as long as he keeps thinking: "If only there were something wrong with my hips. Then everything would come to an end, and my life would find its final and conclusive form," there is not much hope. Resignation is complete. His depression is real, but it cannot be treated in a conventional, medical sense. Life has gone off track, the patient refuses to help himself, and medication is unlikely to be of much help.

> **Female, aged 49 years, with migrating aches.** Thyrotropin normal (not goitre). Swelling around the epiglottal cartilage, in my opinion not corresponding to thyroid gland. Receives physiotherapy, and we agree that the physiotherapist should also massage the neck. We discuss her fibromyalgia, rheumatism, and Sjogren's syndrome, and the patient states that she has "migrating aches" that migrate from one area of the body to the next, like slight cramps. We discuss the nature of such "migrating aches". The patient is referred to a specialist.

Migrating aches are tensions that crawl about in the patient's body like worms. They are a very interesting example of tensions living a life of their own inside the body as a repository of unprocessed feelings. They are not localized in any particular site or organ, but may come and go anywhere in the body, with resulting disturbance of the organ they affect. In our opinion, fibromyalgia, rheumatism, and Sjogren's syndrome are sequelae of such tension.

Biomedicine turns to a molecular analysis of the autoimmune disturbance and, at best, it regards the migrating aches as a rarity, while consciousness-based medicine considers the content of the migrating aches and takes them very seriously. If the patient's subjective complaints can be alleviated, there is a very good chance that the physical disturbances will also pass. Biomedicine rarely succeeds in proper healing of autoimmune diseases like diabetes type I or arthritis; it remains a semi-effective symptomatic treatment.

## CANCER KILLS ONE IN THREE

It is believed that one Dane in three dies from cancer and biomedicine has thus no effective cure for cancer. Since we can live to become 100 years old, many of us will die from cancer around halfway through the life intended by nature.

> **Male, aged 66 years, with prostatism.** Micturition slowly returning to normal, prostate surgery twice and surgery for urethral stricture twice. No pain. Examination: No tenderness corresponding to the bladder. Negative urine stick. Referral to hospital for assessment and treatment.

Prostate cancer, which is what this patient presumably has, is merciful; it grows very slowly and rarely spreads.

## HEART CONDITIONS

It is believed that one Dane in two dies from a cardiovascular disease, so biomedicine has obviously no effective cure against most heart conditions. The general practitioner will see the patient in the community, refer to the hospital, where they are diligently medicated and operated on. Survival statistics on these patients are not impressive. The average event-free survival time is short, often said to be less than 10 years after bypass surgery, where three veins are grafted around the blocked coronary arteries; in the case of three-vessel coronary disease with varying severities of angina and left ventricular dysfunction, adjusted event-free survival (death, myocardial infarction, definite angina, or reoperation) after 6 years were only 23% (one vessel bypassed), 23% (two vessels bypassed), 29% (three vessels bypassed), and 31% (more than three vessels bypassed)[31].

> **Male, aged 52 years, with balloon angioplasty and anxiety.** The patient suffers from anxiety following balloon angioplastic surgery twice [dilation of the coronary arteries with a balloon which is inflated inside the vessel]. We talk about getting rid of the anxiety by accepting it, dwelling on it, perhaps lying in his wife's arms, allowing yourself to be small and afraid — do it a 1,000 times over the next couple of years. His wife is kind and understanding and wants to support her husband. Can return for conversation.

People become afraid when they have heart problems. Years after the problems seem to have been solved, people still tremble with fear. Since half of us die from cardiovascular disease, this anxiety is justified. We really are going to die. And the heart is our Achilles' heel, so to speak. It stops beating, and that is the end of it. Biomedicine has not solved the problem of weak hearts, although enormous progress has been made with for example enzymes that dissolve acute blood clots. Dean Ornish and co-workers[32] have demonstrated that cardiac disease is very sensitive to improved quality of life. His work focused on making the patient “open up the heart physically, emotionally and spiritually”. This is a marvelous project and a very successful one. Only, his colleagues do not really appreciate his work. For how can spiritual openings of the heart do away with coronary stenosis?

## PSYCHIATRIC DISORDERS

Statistically, the incidence of severe mental diseases in the Nordic countries is about 13% (numbers from Norway[33]) and about one in five will at some point in life receive psychiatric treatment with psychotropic drugs. Generally, people who become mentally ill do not recover completely, but people who have a reasonable life at the onset of their illness will often achieve sufficient symptom relief to resume their old lives after treatment.

The situation is different for people who become mentally ill before they have settled down, i.e., when they are young. A mentally ill and unstable person will find it difficult to attain a life. The many recovery studies indicate that only one in five patients diagnosed with schizophrenia attains a normalized existence; the rest of these patients have so many psychotic symptoms throughout their lives that a psychiatrist will still call them schizophrenic. Biomedicine removes some of their symptoms, but not the actual illness. Institutionalization in psychiatric wards teaches them that they need not take responsibility for their survival. Our conclusion is that we need a new psychiatric approach that is better at making the patients well. We need a psychiatric system that understands the actual cause of the psychiatric disorders and treats them on that basis. We will provide our view of such holistic theory below. For the moment, suffice it to say that when patients discover themselves and their purpose in life and learn to be true to themselves and live accordingly, it appears that they actually can become well or cope with life.

> **Male, aged 60 years, with depression.**
> 1. Patient scores HDS 20 (MIES24) on the Hamilton scale, corresponding to depression. Prescribe antidepressants. The patient has presumably been depressed for years, and has been advised not to expect any major improvement for weeks or months.
> 2. The patient suffers from chronic muscular pain, which may be a manifestation of the depression.

Quite frankly, the psychiatric biomedical program against depression and psychosis is not working well. Depression and psychoses may be alleviated temporarily by means of psychotropic drugs, and patients may return more or less to who they used to be, albeit perhaps a little more timid and inhibited. But surely life does not intend us to remain the way we are as people at our current stage of development and then deteriorate physically and mentally over our adult lives? Is it not the meaning of life that we should develop, become better and more alive, and get to know ourselves better?

An episode of depression is an opportunity to take a closer look at ourselves and learn lessons through questions such as: why are my shoes not comfortable to wear? What is it I feel about myself, the people around me and life in general that gives me this unsatisfactory life? Any patient who patiently and laboriously takes on the task of sorting out his own philosophy of life will, in our experience, be richly rewarded for the effort.

## DIFFICULT MEDICAL CONDITIONS

Sometimes patients suffer from something rare and strange. The body is a highly complex structure and any disturbances may take on quite strange, special, and unexpected forms of expression. The poorer the understanding of a patient’s disease or condition, the more difficult it is to treat. Where should you begin and where should you end? Patients with rare diseases are usually referred to specialized units at the hospital, where they are transferred from one unit to another, until somebody feels competent enough to treat them. Having a rare disease may be life threatening. Patients with more common, but incurable diseases (like cancer) also end up in the hospital, but here the purpose is palliative in order to make death as painless and "respectful" as possible. In the hospital setting that is not always possible, where the focus is on acute medicine, which is the reason why many medical centers have created hospices, where people with incurable disorders can die with some dignity. With holistic medicine, both situations may be within the physician’s therapeutic reach, if only the patient is willing to work on himself.

> **Male, aged 44 years, with purpura.** Patient has breathing difficulties, dizziness with headache, swelling around the eyes, sometimes feels very ill, very tired for a long period, partially far-away sensation in the head. Examination: BP 135/85. Weighs 92.5 kg — usually weighs between 55 and 59 kg. Small red patches that do not disappear on pressure! /Purpura [a dangerous rash]/ /suspected immunological disorder. New appointment when we have the results of various blood tests.
>
> Blood test results together with the clinical purpura indicated systemic [involving the entire body] disease, which should be assessed by specialists at the hospital. Referral.

This is a very dangerous situation for him. Purpura — the image formed by thousands of microhemorrhages in the skin — is not to be taken lightly. If he also has microhemorrhages (small bleedings) everywhere in his internal organs, his life is at risk. The condition is difficult to treat pharmacologically, and the outcome of such “immunological collapse” may be death in spite of the greatest expertise. We are not quite as good at adjusting imbalances of that kind, as is generally assumed.

## STAGNANT EXISTENCE OR BURNOUT

Stagnation is an odd phenomenon. People lose the spark of life or they have no purpose, their entire existence and all their human relations decay. In the end, they have absolutely nothing of value, and although their bodies are strong and healthy, they display numerous symptoms, reflecting repression to the body of their emotionally painful lives.

> **Female, aged 37 years, with typical “stagnant” picture.** Presents with distal phalanx of the right second finger, which feels sore and “inflamed” on one side. Examination: Slight redness and tenderness corresponding to the phalanx, but unlikely to be rheumatoid arthritis or other well-defined arthropathy. Additionally: All phalanges of the digits bilaterally “rigid”, cough, tenderness corresponding to the trigger points in arms and legs, severe tension in the neck, back problems — but the knee problems claimed by the patient are unlikely. The cause of the patient’s complaints appears to be tension rather than inflammation. We discuss it: “I have never been able to relax”, “I don’t like just sitting, then I start feeling agitated,” the patient says. EXERCISE in relaxation: “Sit down for 10 minutes with an egg-timer and just sit there without doing anything at all, sense how you feel. Preferably combined with massaging of the many sore muscles. Should return if the problem persists, possibly physiotherapy.

People who do not work on themselves at all or who are totally unwilling to confront their problems in life easily become stagnant. The stagnant picture includes the following aspects: “trouble” in the joints, sore muscles, dizziness and mental clouding, mixed-up human relations, and a distinct lack of initiative and direction in life in general. They somehow appear “clumsy”, “untidy”, and poorly presented, as if they basically refuse to present themselves as people with goals in life and a meaning to their existence.

The clay that should be molded remains unmolded. It is as if they totally lack creative and constructive spirit. Neither medication nor physiotherapy can do much about that. They need to talk about it.

> **Female, aged 46 years, with possible burnout.** Constantly tired, dizzy, impaired concentration, perhaps a slight temperature, throat complaints for about 45 days. BP 130/70. Auscultation of the lungs: slight basal crackles. Throat: still slightly red, no coating. Socially: no longer happy about work. Strict boss who “forbids anything good”, is not allowed to do anything, works too slowly, “the other bookkeeper is much better.” /Suspected atypical pneumonia/ /suspected burnout/ Prescribe Abboticin [erythromycin]. If no marked improvement within 2 weeks, the patient should return to the clinic.

In this case, the approach was simply to prescribe the best drug that we could find for her. Then we wait to see whether the problem might disappear by itself. We hope so, but do not believe it will, although the suspected pneumonia could make the difference. It will probably take a lot more than antibiotics to get her back on her own two feet considering her complaints. We believe that she has a burnout and in need of comprehensive rehabilitation. But sometimes we are tricked. If she does, in fact, have pneumonia, she and her negative attitude might recover completely with the medicine.

## OLD AGE

Old age is one of the strangest phenomena, because there is often a substantial difference between chronological age and physiological age. Young people may appear very old and tired — worn out and incoherent — while old people may appear extremely energetic and fit. Physiological age is determined by our personal energy level. In turn, this is determined by how much of our vital energy is free and how much of it tied to blockage and traumas. At the cellular level, the cells are forever young — they have eternal life — after all, they are 3,800,000,000 years old by now: the cells have always existed almost back to the beginning of the planet Earth, they renew themselves by division, so in principle they never become old. Therefore, it is extremely difficult to perceive age as anything but an energy problem. There is another factor, however, the life purpose[5], which may be fulfilled so that the person feels genuinely full of days. Unfortunately, hardly anybody has succeeded with *that* project in our time. Physiological old age is therefore to a great extent a result of accumulated inner conflicts and susceptible to holistic treatment — people may actually “become 5 or 10 years younger” following 6 months of holistic therapy.

By contrast, biomedical pharmaceuticals bind further vital energy by disturbing the body in all sorts of ways, so that although the symptom addressed by the treatment may become milder, the general health and well being can in fact deteriorate. For that reason, elderly people should preferably not receive medication; nevertheless many elderly people have ten different kinds of pills in their medicine cabinet instead of three kinds at the most, which their bodies can tolerate.

> **Female, aged 56 years, growing old much too soon.**
> 1. Has slept on her side, pain corresponding to outside of left arm for last 3 weeks. Loss of strength assessed as being of “protection – fixation type.” No sensory deficit, no affliction of feet or lower legs /to be followed up/.
> 2. Oedema around the ankles. Prescribe Furix [furosemide].
> 3. Patient requests a blood sample for gout, but there is no physical sign, so there is no immediate indication for it. “If she doesn’t get it, her husband will tear the whole clinic apart.” She is informed in detail of the risk of prescribing too much medicine, if blood tests are not clinically justified and show false-positive results.
> 4. Productive morning cough for many months. Auscultation of the lungs: nothing abnormal discovered. No fever. May have slight bronchitis in spite of the normal examination.

5. We talk about her everyday life, which is difficult; she becomes increasingly insecure. We talk about anxiety and menopause.
6. Headache almost daily. BP 130/90.

This patient has grown old 20 years too soon. She desperately wants to be examined, since there must be a disease, which the physicians have overlooked and for which she can be treated. But no, there is no disease. A good physician often knows intuitively whether or not people are seriously ill. To the best of our knowledge, this patient is not ill. We do not want to examine her for something that we are certain she does not have — with the risk that the blood samples show a slight imbalance. Blood samples often shows false-positive results, some say one in twenty, but sometimes it is much more: compare, for example, the high rate of false positives in blood donor screening for antibodies to hepatitis C virus[34]. So a fine rule is only to test when you suspect a specific disease. All the biomedicine in the world cannot save her. She has to save herself. Otherwise it will not happen.

**Male, aged 79 years with dementia**
1. Vision and hearing no longer good. Should have an appointment with ophthalmologist and audiometry.
2. Dandruff and dry facial skin. Should use a rich skin cream daily on the face and anti-dandruff shampoo.
3. Very dizzy. BP 160/115. Probably drinks far too little, which may also be a predisposing factor of urinary tract infection that he sometimes suffer from. The home care should make sure that he drinks at least 2 litres daily. In addition, slightly confused, possibly also slightly demented. Cannot place the hours on the face of a clock.
4. Still pain in the locomotor system. Nobligan [opioid analgesic], 50 mg capsules, as required, maximum four times daily in addition to regular medication twice daily.
5. Urinary tract infection. The urine sample today negative, excluding 2+ for blood.

Dementia affects a great many elderly people and especially people around them. Several alternative treatments have been developed to work toward the prevention of dementia by psychosocial measures. The idea is to use empathic communication in order to make the patient feel useful again. By contrast, biomedicine currently has no remedies for dementia. Alzheimer Disease (AD) was first described by Professor Alois Alzheimer, Germany, in 1906, when he reported the case of Auguste D, a 51-year-old female patient, he had followed at a Frankfurt hospital since 1901 up until her death on April 8th, 1906. Even after her death, he went on to study the neuropathological features of her illness. Shortly after her death, he presented her case at the 37th Conference of German Psychiatrist in Tubingen on November 4th, 1906 in which he described her symptoms:

- Progressive cognitive impairment
- Focal symptoms
- Hallucinations
- Delusions
- Psychosocial incompetence
- Neurobiological changes found at autopsy: plaques, neurofibrillary tangles and artherosclerotic changes

These symptoms are still the characteristics of AD today, which is the most common cause of dementia in western countries. Clinically, AD most often presents with a subtle onset of memory loss followed by a slowly progressive dementia that has a course of several years. The duration of AD can be 3–10 years from diagnosis to death[35].

## DISCUSSION

The fine art of medicine is to give the patient what he or she needs to get well and healthy. For many reasons, the drugs do not always help and it is therefore important for modern medicine to understand which patients will benefit and also where it will be a waste to give drugs. Many modern drugs have a NNT (Number Needed to Treat) of 2 or more[36,37], and this situation is interesting because it means that only some of the patients will be helped. Therefore the task of the modern physician will be to know which one to treat with a drug and which patient not to treat biomedically, but with manual medicine or consciousness-oriented holistic medicine instead.

When a drug's NNT is 2 or more, and you have no specific reason to believe that the drug will help a specific patient, it means that the patient has a likelihood of only 50% to be helped by the drug; if the NNT is 5, the likelihood is only 20%. But any physician worth his salt wants to cure the majority of his patients. So just using a drug with an NNT of 2 is not good enough and he is forced to use another toolbox. In general, treatment with a drug of NNT higher than 2 can never stand alone, and if the NNT is 5 or higher, an alternative toolbox must desperately be sought. Interestingly, consciousness-based medicine seems to be able to help most of the patients who understand the path of personal development, if the physician masters the art of "holding" and processing, and has the love for his patients necessary to gain the trust needed for the patient to receive the holding.

Another important aspect is that the pharmaceutical industry could be much better by letting the physician and his patient know the NNT number for various drugs, which should be placed on every package. In addition, the pharmaceutical industry could and should do much more research to determine which groups of patients are likely to be helped by the drug[38]. Most drugs work better when a person is otherwise healthy, young, cooperates with the treatment, and is motivated to take the drugs. It is also important that the patient believes in biomedicine, has an orderly personality to keep a high compliance, has good personal networks, employed, etc. So it is very important to include different kind of patients in a drug study and to let us know the NNT in every case. We believe that this should be regulated by law, as the companies have an obvious interest in widening the group receiving the drug, while the physician and his patients have the completely opposite interest. We need to know and find out when to give and take the drug, and when to use alternative medical toolboxes.

So understanding where the biomedicine is likely to work and not to work is the most important issue in today's medical practice. Blindly administering drugs with high NNT numbers to our patients is not going to help much.

## CONCLUSION

The biomedical paradigm is dominating our medical education in most western countries. The modern physician is using pharmaceuticals as his prime tool. Unfortunately, this tool is much less efficient than you might expect from the biochemical theory. The naïve belief in drugs as the solution to health problems of mankind, overlooking important existing knowledge on quality of life, personal development, and holistic healing seems to be the main reason why approximately every second citizen of our modern societies is chronically ill.

The biomedical paradigm and the drugs are certainly useful and in many situations we could not do without the drugs, e.g., the antibiotics curing syphilis and pneumonia, but curing infections in young age is not without consequences as the way we perceive health and medicine is influenced by such experiences. When we get a more severe disease in midlife, we also often believe that a drug exists that can make us healthy again. But now the drugs do not work anymore because we have turned older and have lost much of the surplus and personal energy that made us heal easily when we were younger. Now we need to assume responsibility, take learning, and improve our quality of life. We need a more holistic medicine that can help us back to life by allowing us to access our hidden resources.

Whenever NNT is 2 or higher, the likelihood of the drug to cure the patient is less that 50%, which is not satisfying to any physician. In this case, he must for ethical reasons try something more to cure his patients; this is where the tools of both traditional manual medicine and the tools of a scientific holistic medicine are helpful.

The modern physician cannot rely solely on drugs; he must also have holistic tools in his medical toolbox. With every patient, he must ask himself what he truly believes will help this patient, and this is the line of treatment he must follow. This is the only way we as physicians can improve the general health of our populations. Drugs alone will not do the job.

## ACKNOWLEDGMENTS

This study was supported by grants from IMK Almene Fond. The quality of life research was approved by the Copenhagen Scientific Ethical Committee under number (KF)V.100.2123/91.

## BIOSKETCHES

**Søren Ventegodt, MD,** is the Director of the Quality of Life Research Center in Copenhagen, Denmark. He is also responsible for a Research Clinic for Holistic Medicine in Copenhagen and is a popular speaker throughout Scandinavia. He has published numerous scientific or popular articles and a number of books on holistic medicine, quality of life, and quality of working life. His most important scientific contributions are the comprehensive SEQOL questionnaire, the very short QoL5 questionnaire, the integrated QOL theory, the holistic process theory, the life mission theory, and the Danish Quality of Life Research Survey, 1991–94 in cooperation with the University Hospital of Copenhagen and the late pediatric professor Bengt Zachau-Christiansen. E-mail: ventegodt@livskvalitet.org. Website: http://www.livskvalitet.org

**Mohammed Morad, MD**, is Specialist in Family Medicine, Lecturer in Family Medicine at the National Institute of Child Health and Human Development, Division of Community Health, Ben Gurion University of the Negev and the Medical Director of a large area clinic in the city of Beer-Sheva. He has publications on Bedouin health, health aspects, spiritual health, and aging in persons with intellectual disability, and is a presenter on topics such as health policy and services for the disadvantaged at national and international conferences. E-mail: morad62@barak-online.net

**Eytan Hyam, MD**, is a specialist in family medicine and the Director General of the Soroka University Medical Center, Clalit Health Services, Faculty of Health Sciences, Ben Gurion University, Beer-Sheva, Israel. E-mail: eytanh@clalit.org.il

**Joav Merrick, MD, DMSc,** is Professor of Child Health and Human Development affiliated with the Zusman Child Development Center, Division of Pediatrics and Community Health at the Ben Gurion University, Beer-Sheva, Israel; the Medical Director of the Division for Mental Retardation, Ministry of Social Affairs, Jerusalem; and the Founder and Director of the National Institute of Child Health and Human Development. He has numerous publications in the field of child and human development, rehabilitation, intellectual disability, disability, health, welfare, abuse, advocacy, quality of life, and prevention. Dr. Merrick received the Peter Sabroe Child Award for outstanding work on behalf of Danish Children in 1985 and the International LEGO-Prize ("The Children's Nobel Prize") for an extraordinary contribution towards improvement in child welfare and well being in 1987. E-mail: jmerrick@internet-zahav.net. Website: www.nichd-israel.com
